# Remote Health Monitoring Systems for Elderly People: A Survey

**DOI:** 10.3390/s23167095

**Published:** 2023-08-10

**Authors:** Salman Ahmed, Saad Irfan, Nasira Kiran, Nayyer Masood, Nadeem Anjum, Naeem Ramzan

**Affiliations:** 1 Department of Computer Science, Capital University of Science and Technology, Islamabad 44000, Pakistan; nayyer@cust.edu.pk (N.M.); nadeem.anjum@cust.edu.pk (N.A.); 2 Department of Information Engineering Technology, National Skills University, Islamabad 44000, Pakistan; saad.khan@nsu.edu.pk; 3 School of Computing, Engineering and Physical Sciences, University of the West of Scotland, Paisley PA1 2BE, UK; nasira.kirn@uws.ac.uk (N.K.); naeem.ramzan@uws.ac.uk (N.R.)

**Keywords:** remote health monitoring system, smart health, health edge computing, health fog computing, elderly people health monitoring, ambient assisted living healthcare, concept drift in healthcare, machine learning in healthcare

## Abstract

This paper addresses the growing demand for healthcare systems, particularly among the elderly population. The need for these systems arises from the desire to enable patients and seniors to live independently in their homes without relying heavily on their families or caretakers. To achieve substantial improvements in healthcare, it is essential to ensure the continuous development and availability of information technologies tailored explicitly for patients and elderly individuals. The primary objective of this study is to comprehensively review the latest remote health monitoring systems, with a specific focus on those designed for older adults. To facilitate a comprehensive understanding, we categorize these remote monitoring systems and provide an overview of their general architectures. Additionally, we emphasize the standards utilized in their development and highlight the challenges encountered throughout the developmental processes. Moreover, this paper identifies several potential areas for future research, which promise further advancements in remote health monitoring systems. Addressing these research gaps can drive progress and innovation, ultimately enhancing the quality of healthcare services available to elderly individuals. This, in turn, empowers them to lead more independent and fulfilling lives while enjoying the comforts and familiarity of their own homes. By acknowledging the importance of healthcare systems for the elderly and recognizing the role of information technologies, we can address the evolving needs of this population. Through ongoing research and development, we can continue to enhance remote health monitoring systems, ensuring they remain effective, efficient, and responsive to the unique requirements of elderly individuals.

## 1. Introduction

Remote Health Monitoring Systems (RHMS) can manage, maintain and monitor a specific set of tasks efficiently over a network with reduced cost and errors. This network can be an Internet-of-Things (IoT) system or a local network system with a series of connected devices [1]. They are scalable and provide multiple opportunities to implement changes. They use certain devices, either vision-based devices such as cameras or sensor-based devices such as Accelerometers or Gyroscopes, to form a network. The selection of devices for these systems depends on the environment, requirements, and application [2].

Remote Monitoring System (RMS) is often used in sensor-based technologies with applications such as radars, satellites, and aeroplanes. In the context of this study, another impactful application of RMS is in the health sector, usually labelled as RHMS. Real-time health monitoring of patients by a doctor from a remote location has a significant impact on the avoidance of irregularities. It provides first aid within the nick of time. These systems show great promise, especially for elderly and physically disabled patients [3].

Wearable sensors and health-monitoring devices, including heart rate sensors, pulse sensors, oxygen sensors, and blood pressure sensors, play a crucial role in remote health monitoring systems, whether in open or closed environments, for observing patients [4]. These sensors detect any abnormalities in the patient’s behavior, prompting immediate action from caregivers or doctors, enabling them to take necessary measures promptly to address the situation. In addition to these wearable sensors, vision-based sensors are also employed to monitor the health conditions of patients. For instance, a camera mounted near the patient’s vicinity keeps track of the patient’s movement. If the system detects any abnormal movement by the patient, it promptly triggers an alarm to notify the caretaker [5]. By combining wearable and vision-based sensors, healthcare providers can comprehensively monitor and respond to the well-being of patients in real-time.

In recent years, we have not found any surveys in the healthcare domain that summarize the different computing platforms used in remote healthcare applications. Additionally, a comparison of a variety of Machine Learning approaches has been conducted to identify health-related diagnoses and activities. The primary objective of this study is to comprehensively review the latest remote health monitoring systems, with a specific focus on those designed for the elderly. Moreover, this paper identifies several potential areas for future research like concept drift, which hold promise for further advancements in RHMS.

### 1.1. Data Analysis in the Context of RHMS

One aspect of RHMS is the availability of a considerable amount of data that must be analysed and exploited to attain certain results. Many of these applications usually run on web servers and require continuous transmission and reception of data, which causes a delay in the service; and when it is concerned with real-time monitoring of patients, this delay can be impactful to life-threatening scenarios. To avoid this persisting problem, an efficient remote health monitoring system is analysed based on a layered structure. The layers usually comprise edge computing, fog computing, and cloud computing [6].

### 1.2. Fog and Cloud Computing

There is no need to manage and maintain local or online servers for data management and processing. Cloud servers are used to handle and compute the data over the internet, which reduces the cost and increases the efficiency of the RHMS. Cloud-based monitoring enables effective remote monitoring and smart resource scheduling by removing the delays and data communication issues [7]. Many cloud based health monitoring systems have been presented to overcome the limitations of manual server-based data communication [8]. Though there are many advantages to migrating to cloud-based servers, there are also some concerns. The vast distance between multiple devices can cause high latency in data communication, which can cause problems for IoT apps that require low latency. Security and privacy are also major concerns as the data is globally communicated through different channels along with other users, it may cause data loss and be prone to cyberattacks [9].

Fog computing is an extension of cloud computing, though both are composed of different nodes, which ultimately link to the devices; however, the nodes in fog computing are closer to the end-user or devices as compared to cloud computing nodes. In general, cloud computing can be described as a centralized system while Fog computing is more of a distributed system. Fog computing is not a separate or independent system, but a mediator between device and cloud server, which handles the flow of data from the devices to the cloud. The decision regarding the data and its magnitude to be sent to the cloud server is extremely critical and enables the cloud server to work efficiently and maintain the load between the client and server [10]. Abundant work is being conducted by utilizing the fog computing framework in healthcare monitoring as well. Vora et al. [11] presented a fog computing-based health monitoring system for ambient assisted living. Movement patterns of patients are collected through inertial sensors and the data are passed through fog gateways. The major contribution of this work is the low data communication latency and data load management between the cloud server and output devices. In another work, Vora et al. [12] proposed architecture for remote monitoring of heart rate of patients using fog computing. They used a heart rate sensor to gather data from multiple patients with heart diseases and sent it to the fog layer. They introduced a data compression technique for efficient bandwidth utilization at the fog layer. The results showed better accuracy compared to other state-of-the-art cloud-based architectures.

Though fog computing improves the overall communication between client/server data transmission, it has some drawbacks. With the addition of an extra layer (fog computing layer), the overall system becomes a little complex. Cost is also increased to implement software and hardware utilities and another important consideration is that fog computing is not as scalable as cloud computing [13].

Mobile Edge Computing (MEC) [14], is a distributed computing architecture that brings computational capabilities and resources closer to the edge of the network. It aims to reduce latency and improve the efficiency of mobile networks by deploying computing resources, such as servers, storage, and networking equipment, at the edge of the network, typically at the base stations or access points. MEC [15] enables data processing, analysis, and storage to be performed locally, reducing the time it takes for data to travel back and forth to centralized cloud data centers.

### 1.3. Human Activity Recognition in RHMS

There is a significant focus on ambient assistive living because of a rapid increase in the aging population and related complicated challenges. The goal of improving health care services while reducing the cost is on the agenda of every government. Due to a rapidly increasing aging population and its associated challenges in health and social care, ambient assistive living has become the focal point for both researchers and industry alike [16,17,18].

As mentioned earlier, the current availability of huge data covering every aspect of elderly people’s life is not a big issue. Therefore, once the data are available, the next important component of the remote health monitoring system is its analysis to recognize patients’ behaviour and activities. The automatic interpretation of human activities can play a pivotal role to revolutionize various routine activities. Human activity recognition has been deemed as one of the quintessential research areas for the past two decades [19]. Activity recognition refers to an ability to infer ongoing activity by processing raw data through diversified mechanisms, varying from traditional statistical measures to advanced data mining, machine learning and deep learning concepts [20,21,22,23].

Human activity recognition systems are beneficial in inferring human activities for providing feedback to take necessary actions for intervention. Typically, human activities are classified into two broad categories: (i) ambulation or fitness activities and (ii) functional activities [2]. Uncountable motions, such as walking, jogging, walking in upward/downward directions, fall under the category of ambulation or fitness activities. Functional activities include routine tasks such as attending calls, washing foods, and cooking [20,24]. These behavioural or functional activities can play a consequential role in determining human wellness [25,26]. Human motions of the same activity may have a significant difference because of constraints including environment, time, and culture [27]. Automatic recognition of human activities becomes a challenging task due to diversity in humans’ physical appearances and actions.

### 1.4. Structure of the Paper

This paper provides a comprehensive survey of the remote health monitoring system, especially in the context of elderly people. To this end, this paper discusses various gateways ranging from smart devices to fog/edge computing to cloud-based solutions. Moreover, the paper also discusses automated human activity recognition techniques that provide the basis for doctors and experts to make decisions.

The rest of this paper is organized as follows: Section 2 details the review of gateways often used in remote health monitoring systems, Section 3 provides the details of human activity recognition in the context of the monitoring of elderly people, Section 4 lists existing challenges related to the remote health monitoring systems, and finally, Section 5 provides conclusions.

## 2. GATEWAYS: Data Computation in a Remote Health Monitoring System

Remote health monitoring systems help patients reduce the number of visits to the hospital and predicts health-related anomalies sooner with recent Fog enabled healthcare technologies [28]. The most used sensors in health applications include respiratory rate, heart rate, blood pressure, body temperature, blood glucose, electrocardiogram (ECG), and an electroencephalogram (EEG) [29,30].

In IoT and cloud-based architectures, cloud servers store and process a massive amount of data collected from sensor nodes. Therefore, applications deployed on cloud servers can benefit from large amounts of resources and computation power [31]. Regardless of the benefits that we achieve from this architecture, a cloud-based approach is not suitable for health applications because of high network bandwidth requirements, latency, and scalability issues [32]. Healthcare applications are latency-sensitive; therefore, to design an effective real-time application, the fog-based approach plays a beneficial role [33].

In healthcare applications, wrong treatment decisions could be declared with an increased probability of processing and transmitting large and complex data [34]. Many articles included in this survey discuss scalable solutions for health applications using fog architecture, enabling real-time analysis and decision making based on local network resources [35]. In critical healthcare applications, distributed computing on the fog layer on the edge of the networks ensures low latency, reduced energy consumption, low execution time, scalability, privacy, and security [36].

In rural areas, where remote health monitoring of patients is more challenging because of weak internet coverage, fog computing is a workable solution due to its lower latency and spare capacity of locally available resources. In case of unavailability of the Internet, most critical tasks can be performed on the patient’s data at the fog nodes and forwarded to the cloud later upon internet availability [37].

Mach and Becvar [38] discussed some other data computation architectures such as Mobile Cloud Computing (MCC) and Mobile Edge Computing (MEC). The authors discussed the critical challenges in MEC, i.e., (i) computation offloading to User Equipment (UE) to reduce energy consumption and execution delay, (ii) efficient allocation of the computing resources to minimize execution delay and balance load of both computing resources and communication links, and (iii) mobility management for the applications offloaded to the MEC guaranteeing service continuity. This paper also discussed the computational offloading possibilities including fully offloading, partial offloading, and non-offloading of MEC applications.

Wang et al. [39] discussed fog computing real-time processing and event response for healthcare applications. Their experiments proved that the healthcare system using fog computing responded faster and is more energy-efficient than cloud-only approaches. For example, fog computing can efficiently detect falls of stroke patients by scheduling analytical tasks to the most appropriate edge server, guaranteeing the low latency and high throughput.

Rahmani et al. [40] proposed a smart e-Health system based on a fog computing-based remote health monitoring architecture that uses gateway resources to improve processing efficiency of health sensors’ data. The author showed an RHMS with Early Warning Scores (EWS) using hierarchical fog-assisted cloud computing. The results show improvement in sensing-to-actuation latency in fog computing up to 140ms over cloud computing.

Stantchev et al. [41] illustrated three-level architecture to emphasize the essentials of the computing paradigm related to fog computing to provide improved performance. First, before accessing the cloud, the sensing devices connect to localized fog devices that cater to their needs, such as computing and storage. Fog devices can provide local management for the sensors and handle mobility. Second, interconnectivity with enhanced Quality of Service (QoS) of this computing paradigm as latency is toned down because of the proximity between the fog device and sensors. Third, fog devices provide computing redundancy and backup if the link to the cloud is faulty. In addition, access control can provide better management measures for data flow to and from the cloud. Ko et al. [42] discussed issues in conventional design for mobile computation offloading in which a computation task is fetched to another server only when it is handed over. This mechanism requires excessive fetching of a large volume of data for handover and thus brings long fetching latency. Moreover, it also causes heavy loads on the MEC network. The proposed solution handles this issue by pre-fetching parts of future computation data to potential servers during the server-computation time, referred to as live pre-fetching. This technique can significantly reduce the handover latency via mobility prediction and enable energy-efficient computation offloading by enlarging the transmission time. However, it also encounters several challenges, with the two most critical ones described as follows: the first challenge arises from trajectory prediction. An accurate prediction can allow seamless handovers among edge servers and reduce the pre-fetching redundancy. The second challenge lies in the selection of the pre-fetched computation data. The computation-intensive components should be pre-fetched earlier with adaptive transmission power control to maximize the probability of complete offloading of data from edge devices.

Saidi [43] focused on extending elderly home care in the safest possible conditions by preventing the risks of people living alone. The proposed system monitors older people remotely and designs a method for solving the privacy protection issues for healthcare data based on the F2C computing scheme.

Jamil et al. [44] discussed that increasing numbers of Internet of Everything (IoE) devices are generating vast amounts of data, due to which cloud computing is unable to meet real-time applications such as low latency, location awareness, and mobility support.

Fog computing has overcome these limitations and emerged as a new computing paradigm that complements cloud computing by providing real-time processing and analytics and storage facilities near the edge device. This paper has designed and implemented an optimized job-scheduling algorithm to minimize the delays for latency-critical applications. A case study is discussed for healthcare to demonstrate the latency-sensitive and delay-tolerant requests from IoE devices. The proposed algorithm schedules jobs on fog devices based on the length and reduces the average loop delay and network usage. However, the proposed algorithm reduces the waiting time and can starve the tuples with more extensive lengths.

**Figure 1:**
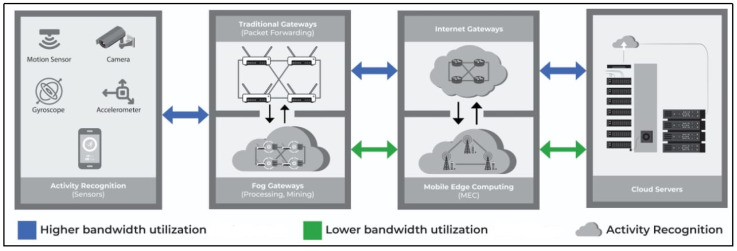
Comparison of cloud based with Fog and MEC.

In Table 1, we identified a set of parameters from the literature to summarize the features and performance of each technology. Next, we explore each of these parameters comprehensively and illustrate how these parameters can affect healthcare applications. In Figure 1, cloud-based sensors data processing is compared with Fog- and MEC-based infrastructure based on lower and higher bandwidth utilization.

### 2.1. Network Latency

Cloud servers are centralized and usually deployed far away from the end-users, so the network latency becomes high. On the other hand, fog gateways are near to end users’ devices, so the network latency is much lower than cloud, as discussed in [40]. The experiment performed by [40] shows that latency in fog is 21 ms and 161 ms in cloud, using the Wifi platform for a healthcare application. In MEC, the user devices connect with the server at the base station, so the network latency is higher than fog.

### 2.2. Internet Bandwidth Utilization

For applications such as remote health monitoring, where health monitoring sensors produce frequent data in large amounts, fog gateways and MEC servers can process and store data locally so that only the summarized data are transmitted over the cloud. Based on this fact, the bandwidth utilization is reduced significantly over the Internet [45]. The bandwidth utilization difference in these technologies’ architectures can be analyzed in Figure 1.

### 2.3. Power Consumption

Performance of FC and MEC is magnificently improved in terms of power consumption over the cloud computing approach. Hartmann [46] discussed that a distributed architecture such as fog consumes less power than MEC and cloud computing. Moreover, for healthcare applications, refining sensor data at the local level reduces the amount of data transmitted over the cloud, which reduces power consumption.

### 2.4. Execution Time

The discussion conducted in [47] concludes that cloud is rich in storage capacity and computing resources. Cloud can also be capable of extending the resources on demand based on the requirements of data processing. Based on this fact, the execution time is very low for processing immense data generated by health applications. Whereas, in MEC, servers with base stations have relatively limited resources, so the execution time to process data is higher. Furthermore, fog devices are usually low-power devices; these devices’ processing power and storage capacities result in higher execution times.

### 2.5. Context Awareness

Context-awareness is one of the essential concepts utilized in health applications where information such as patient location, other patients in the vicinity, and resources in the environment can be exploited. Therefore, collaborations could accomplish real-time context-aware applications such as remote health monitoring among MEC platforms for better decisions. However, since the nodes for fog Computing are usually devices with a narrower view of the network, such as routers or switches, the context awareness is lower than that of MEC. However, the ability to communicate among the nodes mitigates this issue to some extent. On the other hand, cloud computing is low context-aware because it is developed as a standalone server connected to the Internet [48,49].

### 2.6. Real-Time Compatibility

In the cloud computing approach, health sensor data are sent directly to cloud servers without filtering data. Based on this reason, the cloud computing approach is not helpful for real-time applications. In fog computing, local data processing helps health applications to detect unwanted events in real-time and respond to the concerned persons for that event quickly. The real-time feedback collection encompasses solutions aiming to provide quick responses to critical situations in healthcare environments [45]. In [50], the authors implemented a real-time signal processing algorithm for fall detection, delivering information to caregivers. These algorithms are executed at the network’s border by fog servers, collecting and processing all health information. Another approach [51] proposes a real-time analytic system to monitor falls caused by strokes. All mentioned applications need a real-time response to caregivers or actuators for further actions.

## 3. Human Activity Recognition in RHMS

RHMS, which works independently at home and in offices, is now vital in the wake of growing lifestyle-related disease and an increase in the ageing population. Independent of the hospitals, processing and acquisition of the patients’ physiological data is conducted while they are at home or the workplace [52].

Human Activity Recognition (HAR) is a process that involves the recognition, detection, interpretation, and examination of human activities. It mainly focuses on the movements and actions that humans make in their daily lives. The data collected from these actions and movements are collected through different wearable sensors and devices, which can be utilized in several aspects that can make our daily life better and more convenient [20].

Vision-based devices, such as cameras, video recorders, etc., can be fixed in a certain place to track the movements of a person. Over a certain amount of time, enough data can be gathered to identify the movement of a person against a certain action. Similarly, wearable sensors such as motion detectors, compasses, accelerometers, etc., can be attached to a person at different body locations, and movement data can be gathered. The gathered data from these devices is then fine-tuned to remove any redundancies before feeding it to a data-processing model [53].

In recent years, HAR research has attracted significant attention because of its advantages and widespread applications. These widespread applications include (but are not limited to) fashion, smart homes, self-driving cars, surveillance, and healthcare. The ability to recognize and detect human activity has been an important concept in the field of machine learning [27,54,55,56,57]. Different Human Activity Recognition systems have been designed to automate these applications; however, building a fully automated HAR system can be a very difficult task because it requires a huge pool of labeled data and efficient data classification models. Moreover, it is very difficult to accurately classify movement data, as a single activity can be performed in multiple ways, and different activities can be performed in similar ways. Nonetheless, progress is still being made in HAR, as hybrid models are being introduced, which can efficiently differentiate between activities, hence leading to a better classification of features [58].

Activity is recorded using varying modes such as video cameras, RADAR, wearable physiological sensors, device-free sensing such as Wi-Fi, acoustic sensors, etc., [59,60]. The contemporary state-of-the-art has divided these modes into vision-based devices and body-worn sensors [53]. The vision-based devices harness modes, such as a video camera, to capture ongoing activity and are currently being employed by numerous security applications [61,62,63]. However, they often suffer from veracity-related issues due to camera angle, background and, more importantly, they cannot differentiate between targeted objects and other similar moving objects in the area of interest. For instance, a video camera is placed to capture the movement of a patient in a room, but it also captures the activity of people other than the patient [27]. In such scenarios, vision-based approaches may produce inaccurate results for applications wherein criticality must not be overlooked, such as medical health monitoring. On the other hand, wearable physiological sensors mitigate such issues by stipulating correct information with the least involvement of unwanted medium [26]. Wearable sensors like gyroscopes and accelerometer sensors are worn on different parts of the human body to produce 3-axis orientation acceleration, respectively. Besides these, sensors are comparatively less expensive, environmentally friendly, and hold the potential to produce multi-level data resulting in precise information [27].

Presently, ubiquitous sensing that focuses on discovering knowledge from data collected via pervasive sensors is considered a hot area of research [26]. This kind of activity recognition is employed in two different forms, external and wearable sensors [60]. In external sensors, a predetermined point of interest is selected to place devices that capture voluntary interactions among users and sensors [64,65,66,67,68]. At the same time, wearable sensors are attached to the user. Particularly, embedding powerful sensors in smartphones for human activity recognition (HAR) is receiving major attention from the scientific community to meet escalating demands in certain areas, including pervasive and mobile computing, context-aware computing, etc., [26,62,63]. In this survey, we present a comprehensive analysis of the state-of-the-art in the field of human activity recognition in three different dimensions: (1) Evolution of HAR measures from conventional means to machine learning and deep learning, (2) Role of different sensors in remote health monitoring and (3) Strategies to process real-time health data in cloud computing [38,39,40,41,42,43].

With the increasing importance of HAR in IoT and daily life comforts, it is being applied significantly in the Internet of Health Things (IoHT) environment as well [69,70]. HAR is being used to help patients with psychological and paralysis issues. Moreover, HAR is being used for patients with congenital diseases and conditions, especially for children with motor disabilities, to encourage them towards physical activities. HAR is also being used to detect abnormalities in Cardiac patients; it is even used to detect early signs of sickness and illness [71,72,73,74,75,76]. Another aspect of HAR includes the monitoring of elderly patients to detect their physical state. Monitoring elderly patients by attaching sensors to different parts of the body or observing the patient’s movement through a camera can help collect motion data, which can be used to predict irregularities in a patient’s condition, e.g., if the patient has fallen, is standing, is lying, is walking or running, etc. Collecting these data and implementing an interactive method to observe their movements has a significant impact on the health monitoring of elderly patients [77,78,79].

Currently, there are two major ways to acquire human activity data; either it can be accomplished via vision-based devices or through some body-worn sensors. While vision-based data collection has a mature base, it also has some limitations. For example, the lighting conditions of the place, the image quality of the device, the angle of the device, and last but not least, the privacy issue. Whereas sensor-based activity recognition has been initiated in the past couple of years and does not have a mature base, but there are no impactful limitations to it. Over time, different types of sensors, e.g., accelerometer, gyroscope, etc., have been introduced, which also come embedded in most of our smartphones as well. The progress in sensor development has improved to the extent that the issues of magnetic interference in sensor data have also been taken care of. The state-of-the-art sensors can accurately predict the effects of magnetic interference on real-time sensor data. With the rapid development of new and improved sensors, the data collected from them is becoming more and more accurate [25,80,81].

Although the sensors have evolved significantly in the past years, so has been the case with Machine learning as well. The data obtained from vision-based devices or sensors are trained through a Machine learning method to efficiently detect features from the training data. However, the accuracy of these methods depends on the quality of data and the effectiveness of feature extraction. The more labeled the data is, the better the data can be classified, and features can be extracted. Similarly, the better the training model is, the better the accuracy will be. The traditional Machine learning models are available for data classification, but their shortcoming is the manual feature extraction. For better accuracy, Deep learning models such as Convolutional Neural Networks (CNN), Recurrent Neural Networks (RNN), Long Short-Term Memory (LSTM), etc. are used. The solidity of a HAR system depends on the results of data classification of the Deep learning models. Inaccurate classification of features can also lead to detrimental effects for users [82,83,84,85,86].

Human activities are difficult to classify due to their irregularities. Human activities vary from person to person and can be performed in multiple ways, hence making it a challenging task to classify them efficiently; in such a case, HAR using vision-based devices is a more challenging task as it involves various limitations and challenges. The data or dataset collected from a video or pictures may have background lighting issues, camera angle issues, and vice versa. These issues cannot be sorted out by generic means, thus making the system costly and complex. To overcome these hurdles and limitations, in the past few years, sensors have been used to extract movement data with minimum interference of any unwanted medium [87,88,89].

Sensors are less costly, can provide multi-levelled data, provide precise data, and can be used in any environment. However, even a slight fault or malfunction in sensor hardware can cause adverse effects on the collected data; therefore, it is a must to test the accurate working of the sensor before data gathering. Activity Recognition using standard Machine Learning approaches such as Support Vector Model (SVM), decision tree, etc., can produce substantial results in a controlled environment, but these models are strictly dependent on the data because huge datasets require a huge amount of time for training. All in all, the accuracy of these machine learning models strictly depends on feature extraction as the process is not automated [20,90].

Chang et al. [91] proved that SVM, along with conventional Artificial Neural Networks, can achieve acceptable results; however, they still lacked accuracy. Islam et al. [92] proposed a hybrid feature selection model, which used Sequential Floating Forward Search (SFFS) for feature selection and extraction. Best features are selected, from a subset of features, based on certain criteria, and pairs of best features are created and compared with the next subset of features. The overall feature extraction process becomes efficient and optimal features are extracted. An SVM is then used to classify the data. The main contribution of this work is the efficient utilization of the SFFS module for efficient feature extraction. However, the only drawback of this approach is that it performs well if the dataset is relatively small. The performance degrades if the dataset is too large or there are too many redundancies in it. Hence, it can be concluded that the HAR systems using conventional machine learning models usually require preprocessed data to produce convincing results.

Due to the limitations mentioned previously, machine learning has been integrated with Deep Learning techniques, which cover all the limitations in conventional machine learning models and provide a broader aspect for feature extraction. Deep Learning involves in-depth processing of data, efficient feature extraction, and layered structure for better classification. There are no such drawbacks of Deep Learning other than the increased computational cost for complex and increasing amount of data. Deep Learning models are scalable but hard to debug if the algorithm is too complex [93].

In recent years, Deep Learning has achieved remarkable results in the areas of human activity recognition, image recognition, and Natural Language Processing. One of the impactful aspects of Deep Learning is automatic feature detection and data classification with high accuracy, hence leading to a high demand in the field of HAR. Several Deep Learning models have been introduced over the past years, and each one of them has positives and negatives of its own. Deep Learning Models are designed and inspired by the structure of the human brain, which is the reason they are usually referred to as Neural Networks. CNN is a multilayered model, which is used to extract features from data [94]. Their generic structure consists of an input layer, two hidden layers, and an output layer. The input layer consists of the input data, which are fed to the neural network. The hidden layer usually consists of two sub-layers; one is the convolution layer in which multiple filters are applied to the data and the second is the pooling layer, which merges the data from the convolution layer. The output layer is connected to, and is part of, a fully connected layer that consists of the merged data from all the hidden layers and classifies it.

RNN is a gate-based neural network that consists of multiple gated units. The output of each gated unit can be fed an input to the other unit because they can remember the input of other units. RNN is widely used in the applications of Natural Language Processing and Image detection [95].

LSTM is a type and subclass of RNN. The major difference between LSTM from generalized RNN is that RNN suffers from vanishing gradient problems. LSTM, however, covers up all those problems in RNN by introducing Gates. Moreover, they consist of memory cells that can store input data for a longer period, which is very beneficial when the huge pool of data is being trained [96].

These are the most widespread and commonly used classification models used in HAR in recent years due to their exceptional performance and results. Most of these approaches utilize sensor data for training purposes. Nonetheless, several HAR systems use wearable sensors to collect data. These wearable sensors mostly include inertial sensors, such as accelerometers and gyroscopes, because they are also included in smartphones [58,97].

Many activities, such as lying, walking, running, sleeping, and walking, have been identified by different HAR systems to detect the rate of falls in elderly people and to avoid it. But these approaches utilize multiple sensors to collect movement data, which can be a hassle that affects people’s daily lives. Hence, more studies have been conducted that are based on a single sensor, which shows notable results when it comes to basic activities such as walking, running, and sitting but fails when it comes to complex activities such as smoking, dancing, and exercising [98].

To overcome this hurdle, sensors, such as accelerometers or compasses, are used in conjunction with other sensors, e.g., gyroscope, heart-rate sensors, etc. Though the huge pool of data needs to be segmented by having data from multiple sensors, an efficient classification model can achieve higher accuracy when it comes to feature extraction [99,100]. Wan et al. [101] proposed a CNN-based approach, which showed that the classic CNN still outperforms the conventional LSTM, Bidirectional LSTM (BLSTM), MLP, and SVM models when it comes to the accuracy of feature extraction. However, the structure of these models was not optimized, so the results may vary when it comes to precision. The major contribution of these approaches is the significantly increased efficiency by adding a context-aware classification module that overcame certain errors. The results from these approaches showed a significant increase in classification accuracy as compared to other classification models such as Decision table, Random Tree, etc.

Wang et al. [102] demonstrated the comparative analysis of conventional HAR approaches with advanced HAR approaches. Conventional HAR approaches based on pattern recognition collected raw data from devices such as sensors, Bluetooth, Wi-Fi, etc. and the features were manually extracted and input to a certain Machine Learning model for training. However, these methods contain limitations such as only simplistic features could be extracted, which ultimately leads to average performance. Advanced HAR approaches based on Deep Learning models overcame all these limitations by introducing Neural Networks. The manual feature extraction has been fully automated. Moreover, the performance of Deep Learning Methods on unlabeled data also far exceeds conventional Machine Learning methods. The focus of this survey was to demonstrate the importance of evolved Deep Learning in HAR and the need for lightweight Deep Learning models to minimize the cost of complex systems.

Ignatov Andrey [103] proposed an enhanced CNN, which overcame the time-series length effects when extracting features for real-time activity recognition. Testing the proposed system on UCI-HAR (University of California Irvine—Human Activity Recognition) and WISDM (Wireless Sensor Data Mining) datasets, superior results were achieved as compared to the state-of-the-art CNN-based systems. The proposed work was not only superior in feature accuracy but was also low cost. However, the data required for this approach was preprocessed. As there is no noise removal module or auto-labeling module involved, the performance of this approach will not be up to the mark for weakly labeled data.

Zhou et al. [104] designed a semi-supervised deep learning framework that was able to extract features based on weakly labeled data. An auto-labeling system was introduced to label the unlabeled data, which drastically increased the learning accuracy. A distance-based reward rule strategy was used to handle the labeling of unlabeled data. The results of the auto-labeling module were fused with other sensor data and finally passed through a conventional LSTM module for better feature extraction from the data, which ultimately led to an efficient classification. However, this approach required a large dataset of unlabeled data for efficient auto-labeling, thus increasing the cost of the proposed system. Moreover, by using the BLSTM, data could have been more efficiently classified with the availability of a larger dataset.

Xu et al. [105] proposed a hybrid neural network approach (InnoHAR), which combined RNN with Inception Neural Network (INN). INN consisting of various deep layers has multiple convolution layers parallel to pooling layers, which form an inception layer. These convolution layers are a matrix of 1 × 1, 1 × 3, and 1 × 5, respectively. The main idea behind the inception layer is to allow the filters to select the required size itself rather than wasting resources. The output from the INN is then passed through 2 GRU (Gated recurrent unit) layers for better time efficiency. The results tested on 3 public datasets showed better results compared to the state-of-the-art Deep Convolutional and LSTM (DeepConvLSTM) model and CNN model. However, this approach considered the already-available preprocessed datasets and did not experiment on the real-time sensor data, which requires additional modules such as noise removal and data segmentation. However, INN has poor initialization, which requires a lot of computation to get over it, and minor changes to the model require retraining of data, which is costly. A fine-tuned CNN can achieve the same performance, which is the reason INN is not used much in state-of-the-art approaches.

Jiang et al. [106] proposed an Attention-based Bidirectional LSTM (ABLSTM), which used a BLSTM to train data in multi-directions. This approach is based on Wi-Fi data and thus involves the need for BLSTM, which can process the state of signal data before and after processing. Two-layered BLSTM is used with one layer directing the data forward and the other one redirecting the data backward. The output from the BLSTM is passed as input to the Attention Model. The Attention Model focuses on certain features of data that are of interest based on some speculations. These speculations involve the scoring of vector data using the ReLu function and then passing through the Softmax layer for classification. The results showed superior classification accuracy compared to other similar approaches. However, the experiments were based on a single channeled Wi-Fi device without real-time data collection. These two factors can have a huge impact on the accuracy of the proposed system. Real-time data involve multiple types of interferences on the readings, which can be caused due to a certain environment or magnetic interference. The dataset used for this was based on single-user data and the dataset itself was supervised. A lot of work can be further conducted on this approach. There has been a focus on supervised learning in past research because labeled data are not available in abundance; hence, recent approaches mostly focus on semi-supervised data.

Zhu et al. [107] proposed a novel Deep LSTM (DLSTM) approach for efficient feature recognition. Both labeled and unlabeled, data are used to train the model to detect human activities using smartphone sensors. DLSTM involves multiple LSTM layers between the input and output layers. The raw data are passed through the augmentation phase to increase the amount of data and Gaussian noise removal, which is performed to filter any inconsistencies in the data followed by the extraction of low-level features. These low-level features are dropped out and the rest of the features are passed to DLSTM for high-level feature extraction. The unsupervised data loss, which involves unlabeled data, is calculated and labeled based on some predictions. The results of the proposed approach were benchmarked against the UCI-HAR dataset. The results showed its supremacy over other semi-supervised learning methods. However, this approach was performed in a controlled environment. In an uncontrolled environment, where a single activity can be performed in multiple ways or different activities can be performed in a similar way, the results may vary.

In a recent study, Wang et al. [108] proposed a hybrid 1-dimensional approach. Data from multiple sensors are passed through a convolutional neural network and the output is passed to the LSTM module, which classifies the data. The main achievement of this approach is the identification of activity transition along with activities in general. Most of the proposed works do not consider this factor while designing an approach; however, in human behavior recognition, this is an important task. Moreover, activity transition detection has a significant effect on real-time movement recognition. The data from two sensors (accelerometer and gyroscope) are combined into a 2D array and then passed to the CNN. The respective CNN is a three-layered architecture with three hidden layers, each consisting of a convolution layer and a pooling layer. The output from the CNN is passed into the LSTM module in the form of a vector. The features extracted from LSTM are passed to a fully connected layer which undergoes the process of Batch Normalization and is finally forwarded to the Softmax layer for classification. The results of this approach were benchmarked against the publicly available HAPT (Human Activities and Postural Transitions) dataset, which already contains the activity-transition data. The results showed that, not only did this approach have a better activity recognition rate than other Deep Learning models such as CNN, LSTM, CNN-BLSTM, and CNN-GRU, but it also had a better activity transition recognition rate. The limitations of this approach lie in the fact that only basic activity transitions (lying–standing or sitting–standing) were identified. Complex activity transitions, such as walking–smoking, driving–eating or sitting–reading, may be a different case [108]. Moreover, multiple users may have different movements on activity transition while this study is based on a smaller number of people’s movements.

Lu et al. [109] proposed an approach for efficient data classification, focusing on daily life activities. They categorized activities into two types: countable activities, which involve activities with a fixed number and iterations of gestures like walking, sitting, eating, and smoking, and uncountable activities, which involve complex and uncountable gestures like dancing and exercising. Interestingly, in some cases, even walking can be considered an uncountable activity. The researchers used a method called SFFS (Sequential Floating Forward Selection) to select relevant features for data extraction. Furthermore, they introduced three new features to enhance the classification process. Using Sliding Window, nine features were extracted against every activity using the publicly available DaLiAc dataset and self-gathered (AmA) dataset.

The features extracted were based on every activity’s specific patterns. The extracted features were tested on conventional M.L (Machine Learning) models such as KNN, SVM, GBDT (Gradient Boost Decision Trees), and Random Forest. The results showed a huge boost in classification accuracy as compared to conventional state-of-the-art M.L approaches mentioned before. However, there are certain limitations of Sliding Window such as the computational cost. Cost can be minimized by increasing the window size; however, this will affect the accuracy of feature extraction. Moreover, as mentioned before, the same activity can be performed in multiple ways and vice versa and this method does not take this factor into account. Table 2 summarizes the following section to point out the strengths and weaknesses of referenced approaches. In the “Strength” column, the focus is on the positive aspects or advantages of each model. These strengths highlight the key features or functionalities that make a particular model effective for activity recognition. For example, some models may excel in feature extraction, achieving high accuracy, handling long-term dependencies, or outperforming conventional approaches. In the “Weakness” column, the focus is on the limitations or drawbacks of each model. These weaknesses point out the aspects where a particular model may fall short or face challenges. For instance, some models may have limited accuracy, slow network performance, overfitting issues, difficulty in adapting to certain configurations, or high time complexity.

To summarize, vision-based devices like cameras offer tracking, but can suffer from issues and privacy concerns. Wearable sensors provide precise and multi-level data with less cost and interference. Deep learning models automate feature extraction and outperform traditional machine learning models. Multiple sensors improve classification accuracy, especially for complex activities. Semi-supervised learning and context-aware classification enhance efficiency. Overall, wearable sensors and deep learning models show promise in HAR, overcoming limitations of vision-based approaches and manual feature extraction.

## 4. RHMS for Elderly People

The smart healthcare monitoring system is proposed in [52]. This system can highly contribute to providing a comfortable and safe environment for elder and disabled people. This can help them live independently without the fear of any emergency or critical healthcare situation through continuous monitoring of their health. The proposed framework collects and accumulates patients’ physiological data with the help of wearable sensors and transmits them to a cloud server for data analyzing and processing. Hence, any change in a patient’s health data will be detected and transmitted to the patient’s doctor through the hospital’s cloud server. Thus, any detection of disorder in a patient’s data will be reported to the patient’s doctors via the hospital platform. The framework is a simple technology based on a fixable architecture that can be scaled and easily expanded, thus providing stable and cost-efficient systems to monitor elderly patients remotely. In addition, the results show that the system could efficiently contribute to improving healthcare services by being able to monitor the patient’s health in real-time detecting symptoms remotely. The proposed system, which can monitor patients’ symptoms remotely and in real-time, is highly effective.

Having a powerful effect on physical and mental health and robust association with many rehabilitation programs, Physical Activity Recognition and Monitoring (PARM) have been considered as a key paradigm for smart healthcare [110]. Traditional methods for PARM used controlled environments, intending to increase the identifiable activity subjects completely and improve recognition accuracy and system robustness using novel body-worn sensors or advanced learning algorithms. The system has now changed with cost-effective heterogeneous wearable devices and mobile applications. PRAM has been transferred to uncontrolled and open environments. However, these technologies and their results with traditional PRAM are currently less known. To help understand the use of IoT technology in PRAM studies, this research will provide a systematic review, inspecting PARM studies from a typical IoT layer-based perspective. First, it will summarize the modern techniques in traditional PARM methodologies as used in the healthcare domain, including sensory, feature extraction, and recognition techniques. The second thing this research explains is the identification of some new research trends and challenges in PRAM studies in the IoT environment. Finally, this paper includes a few successful studies to inculcate PRAM in industrial applications. In the last two decades, several studies have been conducted to address critical issues of PRAM because of its importance in healthcare support in a variety of chronic diseases, musculoskeletal rehabilitation, independent living of the elderly, as well as fitness goals for active lifestyles. The contribution of this work is from the perspective of the Internet of Things (IoT) that sequentially covers the sensing layer, network layer, processing layer, and application layer, distinctively and systematically summarizing existing primary PARM devices, methods, and environments. Wearable and portable sensors/devices, inertial signal data processing, and classification/clustering approaches are described and compared in the light of physical activity types, subjects, accuracy, flexibility, and energy. Typical research and project applications regarding PARM are also introduced.

In [111], the use of RFID sensors and accelerometers has been proposed to recognize a user’s daily activity. A decision tree is employed to classify five human body states using two wireless accelerometers, and detection of RFID-tagged objects with hand movements provides additional instrumental activity information. The system has already developed tagging and visualization tools, making it widely applicable for caring for elderly people’s health.

Ming et al. [112] proposed a CNN-based elderly monitoring system. The approach utilizes vision-based devices to capture movement data. They ensure sensitive data protection by introducing a key-based authorization module with the CNN. The experimental evaluation reveals the system to be sustainable towards explicit breach attempts. The proposed framework was tested on the publicly available UCI-HAR dataset with six basic and six transitional activities and achieved an overall accuracy of 92.02%.

Yang et al. [66] proposed an RFID-based CNN model for the posture detection of elderly people. Kastersen’s dataset is used in order to evaluate the approach. The dataset consists of 245 action instances for seven different activities over 28 days, sensed using RFID technology. They recorded four daily life activities, which include “brushing teeth, taking a bath, eating and getting dressed”. The CNN utilized was a dense network, which involved dense layers, thus making the proposed model complicated and prone to errors. The model demonstrated an accuracy of 82.78%, which showed the effectiveness of the proposed approach. However, in real-time scenarios, this approach may not produce substantial results.

To provide a comparison between the aforementioned approaches, let us take a closer look. In one approach [52], a smart healthcare monitoring system is proposed, utilizing wearable sensors to collect patients’ physiological data, which is then transmitted to a cloud server for analysis. Changes in the patients’ health data are detected and reported to their doctors, enabling real-time monitoring and detection of symptoms. This scalable and cost-efficient system aims to improve healthcare services for elderly patients remotely.

Another approach [110] focuses on PARM in uncontrolled environments using IoT technology. It provides a systematic review of traditional PARM methodologies, discussing sensory, feature extraction, and recognition techniques. It also explores new research trends and challenges in PARM studies within the IoT environment.

Additionally, RFID sensors and accelerometers are employed in an approach [111] to recognize daily activities of users, using a decision tree for classification. This system combines wearable sensors and RFID technology to provide valuable information about user activities.

A CNN-based elderly monitoring system [112] utilizes vision-based devices and sensitive data protection measures, achieving high accuracy in activity recognition. This approach ensures privacy and security while effectively monitoring elderly individuals’ movements.

Finally, a CNN model for posture detection of elderly people is proposed [66] using RFID technology, demonstrating good effectiveness but potential limitations in real-time scenarios. This system focuses on posture detection, which is crucial for maintaining the health and safety of elderly individuals.

Each of these approaches brings unique features and advantages to the field of healthcare monitoring and human activity recognition, catering to different scenarios and requirements.

**Figure 2:**
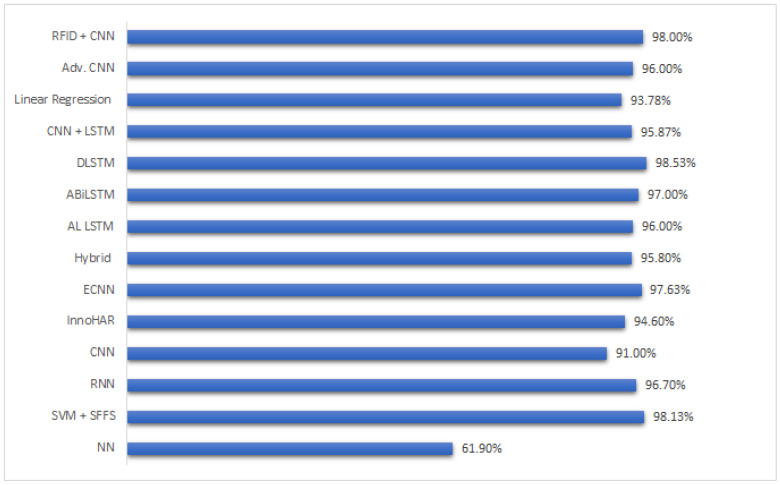
Comparison of Machine learning and deep learning techniques in healthcare system [91,92,95,101,102,103,104,105,106,107,108,111,112,113].

Table 3 summarizes the Section 4 to point out the strengths of referenced approaches. Figure 2 presents a summarized accuracy-comparison graph, which visually represents the information provided in the aforementioned table. The arrangement of the approaches in the graph takes into consideration both the publication year and the novelty of their architectures. Notably, the graph excludes certain approaches from the reference table that exclusively encompassed a systematic review of diverse frameworks. The graph depicts that the mean accuracy of the various approaches lies within the range of 90% to 95%. This range signifies a customary level of accuracy attained through the utilization of neural network architecture. However, it is imperative to acknowledge that the majority of these approaches prioritize enhancing the duration of model training, rather than solely emphasizing accuracy. As expounded upon in the preceding paragraphs, the significance of model training time should not be disregarded, particularly when formulating an approach that concentrates on unsupervised data.

## 5. Major Challenges in RHMS

### 5.1. Data Accuracy and Availability in Real-Time

Logically, the most complex challenge is related to the accuracy of remotely accessed data. Many of these come under scrutiny from patients and medical staff. It is difficult for patients accustomed to traditional methods to trust that a small device will provide great data about their health that they can provide to their doctor. Likewise, front-line medical service providers find it easy to make decisions based on the information obtained through traditional methods. They are also inclined to avoid automated digital systems due to their data inaccuracies. Furthermore, providing information in real-time is also a big challenge. In particular, if data are to reach a doctor’s system from a patient’s device via a mobile network, it must first go to the service provider’s infrastructure and then reach its destination via the internet. In the meantime, if there is a problem somewhere, the data will not be provided. Moreover, mobile networks are not always available. Therefore, if any data on which the patient’s life depends are not available to the doctor at the appropriate time, then the entire RHM system will become meaningless.

### 5.2. Data Security and Protection

Besides the accuracy and availability of the data, its security and protection are also extremely critical. In this case, healthcare standards need to be met. Also, strong data management practices are essential. A large part of the data management is usually handled by third parties, which itself possesses a potential risk on individuals’ data. On the other hand, the challenges for hospitals are also not insignificant, as they risk integrating third-party systems that endanger the safety and privacy of their patients.

### 5.3. Selection of Sensors and Devices

In any RHMS, the highlight is the inclusion of sensors and wearable gadgets. They can be available in a variety of sizes and types. Every sensor may play a vital role in one disease and may become completely irrelevant in some other disease. Therefore, the selection of sensors affects the overall efficiency of the system and is not a straightforward task.

### 5.4. Detection of Concept Drift

In RHMS, machine learning algorithms are used to develop prediction models over Cloud, MEC, fog, edge layers. These models are trained to over historical medical data of different patients to predict falls stroke [113], fall detection [114], and different types of diseases related to health of patient [115]. However, these models are not predicting accurately over a period. This is known a Model or Concept drift in different systems [116]. Many of the drift detection approaches has been proposed in different streams of applications [117,118,119]; however, much less in stream of RHMS.

One recent approach, Ensemble and Continual Federated Learning (ECFL) [120], is a distributed machine learning approach that considers concept drift detection over multiple mobile devices. This approach utilizes ensemble learning, where multiple learning algorithms train models locally and aggregate globally to obtain better predictive performance. In the concept drift detection approach, ECFL preserves the confidence (probability) of predicted labels, data instance values, and the predicted label in a sliding window with each new prediction. The ECFL drift detection algorithm detects changes in two consecutive windows on each edge device. If the change crosses a threshold, the algorithm generates a drift detection alarm. This approach considers the ML model’s confidence level of prediction. However, if the model is not well-calibrated, in case of drift, the model can predict the label with the same confidence as before the drift. In this case, as the confidence value may not differ in case of drift, the detection algorithm may fail to detect the drift.

Under distributed concept drift, the time of change-points of probability distributions can differ across clients/devices [121]. Wang et al. [121] detect the loss in model at each client and develop a cluster on which drift is detected. The retraining is performed for clients that are under the cluster. This approach maintains the accuracy of the overall application developed over the federated platform. However, the measurement of model loss is still uncovered in the scope of this article.

The area of concept drift still has vast space for research to accelerate. The issue of concept drift is critical in RHMS because of inaccurate prediction. More research is needed to improve the detection and handling of concept drift in distributed machine learning systems, especially in the context of real-time healthcare monitoring systems.

## 6. Conclusions

The rapidly growing elderly population and the provision of healthcare facilities to them present a major challenge for governments and healthcare departments. For elderly people, going to hospitals daily to inform doctors about their health and seek advice is impractical. Therefore, Remote Health Monitoring Systems (RHMS) offer a viable solution for both elderly patients and doctors. In this paper, we conducted a survey of literature related to the development of efficient RHMS.

We reviewed existing data sensing and gateway technologies, as well as state-of-the-art Human Activity Recognition (HAR) systems. Our survey explores the benefits of Fog and mobile edge computing, which overcome limitations of cloud computing such as high bandwidth, high latency, and high power consumption. Fog and edge computing have emerged as a new computing paradigm for real-time sensor processing, analytics, and storage facilities near the edge device. The study provides insights for future work, encouraging the use of fog and edge computing in health applications to achieve real-time response to actuators.

Furthermore, we enlisted promising existing RHMS systems, highlighting their potential in healthcare. Finally, we identified and discussed current challenges related to the development of RHMS. Our future work after this survey leads us to address issues related to concept drift in Machine Learning (ML) health monitoring models in the distributed fog environment. Concept drift is a crucial consideration to ensure the accuracy and reliability of real-time health monitoring systems.

In conclusion, RHMS holds great promise in addressing the healthcare needs of the elderly population. By leveraging fog and edge computing, we can enhance the efficiency and effectiveness of health monitoring systems, enabling real-time responses and improving the overall quality of healthcare services for the elderly.

## Figures and Tables

**Table 1:** Gateways comparison with different parameters.

	Cloud Computing	Mobile Edge Computing	Fog Computing
**Network Latency**	High	Medium	Low
**Internet Bandwidth Utilization**	High	Medium	Low
**Power Consumption**	High	High	Low
**Access Mechanisms**	Wi-Fi, Mobile Networks	Mobile Networks	Bluetooth, Wi-Fi
**Execution Time**	Low	Medium	High
**Resources Availability**	High	Medium	Low
**Context Awareness**	Low	High	Medium
**Real Time Compatibility**	Low	Medium	High
**Technology Devices**	Centralized Servers, Data Centers	Servers running in base stations	Gateways (Routers, Switches)

**Table 2:** Summary for Section 3.

Ref.	Model (M.L/D.L/Hybrid)	Strength	Weakness
[91]	M.L (SVM+ANN)	A unique architecture fused basic SVM with conventional ANN. Can be very useful for shallow feature extraction.	Average accuracy, slow network.
[92]	M.L (SVM+ SFFS)	A very efficient lightweight feature filtration technique employing SFFS module.	Shows good results on smaller datasets only.
[94]	D.L (CNN)	Detailed Survey on CNN & its state-of-the-art applications.	CNN cause overfitting, and typical models fail to adapt to certain configurations.
[95]	D.L (RNN)	Can outperform CNN’s in extracting long term dependencies.	Can cause extreme exploding gradients.
[96]	D.L (LSTM)	The memory cell enables the network to perform back propagation and remember long term dependencies, which better correlates data; hence, they outperform conventional RNN’s.	Training time increases exponentially on larger datasets.
[101]	D.L (CNN)	Context-aware classification handled some errors in conventional CNN, which increased the overall accuracy.	Compared with vanilla models with no parameter adjustments or fine tuning.
[102]	M.L/D.L/Hybrid	A detailed survey on conventional vs. advance activity recognition approaches in both M.L and D.L. Portrayed the advanced in D.L.	None.
[103]	D.L (Enhanced CNN)	Showed superior results compared to state-of-the-art works.	Results are based on strongly labelled data only. Performance may vary on weakly labelled data.
[104]	D.L (LSTM+ALM)	Auto labelling module showed significant improvement in accuracy.	The auto labelling module required a large pool of unlabelled data, which made the system costly.
[105]	Hybrid (InnoHAR)	A fusion of RNN with Inception neural networks showed good performance on smaller and larger datasets.	Not implemented in real-time scenarios. Moreover, the configuration of INN is very complicated if a change or update is required.
[106]	D.L (ABiLSTM)	Attention-based BLSTM implemented on Wi-Fi data. Attention module filtered the features-f-interest and dropped low level features, thus making the proposed approach time efficient.	A single channelled Wi-Fi was used without any real-time data collection, which is not a viable source of activity recognition data.
[107]	D.L (DLSTM)	A DLSTM based on labelled and unlabelled data, which extracts high level features and retrains low-level features and labels the unlabelled data. Superior accuracy compared to state-of-the-art LSTM works.	Results generated in a controlled environment; performance may vary in real-time scenarios. Moreover, DLSTM structure makes the network too slow and time complexity increases.
[108]	Hybrid (CNN+LSTM)	A state-of-the-artwork employing postural transition with static activities. Showed superior performance compared to several approaches employing transition activities.	Complex structure only considers basic static and transition activities while the experiments were based on a pre-processed dataset with abundant features. Performance may vary for datasets where number of features is far less.

**Table 3:** Summary for Section 4.

Approach	Type	Strength
[52]	Wearable sensors	Able to detect abnormalities in elderly people and capable of employment in real-time scenarios
[111]	RFID + wearable sensors	By the tracking of hand motion, RFID tagged objects are able to be detected which provides additional pattern data for efficient human activity recognition
[112]	CNN + vision devices	Introduced an authentication-based access network to avoid any unwanted access or breach in the network
[66]	RFID + CNN	A dense CNN with RFID unit brought forward a novel RSS based approach. However no solid strengths were presented in the research work

## Data Availability

Not applicable.

## Abbreviations

The following abbreviations are used in this manuscript:

MDPI	Multidisciplinary Digital Publishing Institute
DOAJ	Directory of open access journals
TLA	Three letter acronym
LD	Linear dichroism
